# Structural and functional investigation of a fungal member of carbohydrate esterase family 15 with potential specificity for rare xylans

**DOI:** 10.1107/S205979832300325X

**Published:** 2023-05-25

**Authors:** Scott Mazurkewich, Karoline C. Scholzen, Rikke H. Brusch, Jens-Christian N. Poulsen, Yusuf Theibich, Silvia Hüttner, Lisbeth Olsson, Johan Larsbrink, Leila Lo Leggio

**Affiliations:** a Wallenberg Wood Science Center, Division of Industrial Biotechnology, Department of Life Sciences, Chalmers University of Technology, 412 96 Gothenburg, Sweden; b Department of Chemistry, University of Copenhagen, Universitetsparken 5, 2100 Copenhagen, Denmark; Station Biologique de Roscoff, France

**Keywords:** lignocellulose degradation, glucuronyl esterases, hemicellulose, α/β hydrolases, biomass conversion, rare xylans, *Lentithecium fluviatile*

## Abstract

In plant cell walls, covalent bonds between polysaccharides and lignin increase recalcitrance to degradation. Ester bonds are known to exist between glucuronic acid moieties on glucuronoxylan and lignin, and these can be cleaved by glucuronoyl esterases (GEs) from carbohydrate esterase family 15 (CE15). GEs are found in both bacteria and fungi, and some microorganisms also encode multiple GEs, although the reason for this is still not fully clear. The fungus *Lentithecium fluviatile* encodes three CE15 enzymes, of which two have previously been heterologously produced, although neither was active on the tested model substrate. Here, one of these, *Lf*CE15C, has been investigated in detail using a range of model and natural substrates and its structure has been solved using X-ray crystallography. No activity could be verified on any tested substrate, but biophysical assays indicate an ability to bind to complex carbohydrate ligands. The structure further suggests that this enzyme, which possesses an intact catalytic triad, might be able to bind and act on more extensively decorated xylan chains than has been reported for other CE15 members. It is speculated that rare glucuronoxylans decorated at the glucuronic acid moiety may be the true targets of *Lf*CE15C and other CE15 family members with similar sequence characteristics.

## Abstract 2

Structural investigation of a presumed fungal glucuronoyl esterase reveals a classical serine hydrolase active site but an unusual ligand-binding site. Functional analysis showed a lack of activity on a wide array of substrates commonly utilized by this family of enzymes. It is hypothesized that this enzyme needs complex plant cell-wall substructures for activity.

## 1. Introduction

**Figure 1:**
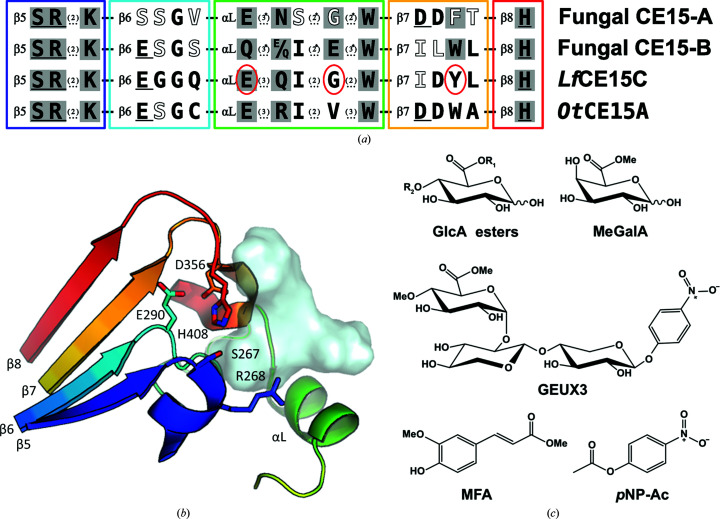
Overview of sequence signatures, structure and model substrates of GEs in CE15. (*a*) Sequence signatures for the CE15-A and CE15-B subgroups as described previously (Ernst *et al.*, 2020 ▸) and the corresponding sequence in *Lf*CE15C, with significant residues differing from the CE15-B signature circled in red. The four signature regions are separated by dashes, while additional residues that are not shown within the regions are indicated by dots with the number of residues in parentheses (asterisks indicate that the number is variable). The location on secondary-structure elements is indicated (see below) and residues expected to directly contact the substrate are shaded. Fully or almost fully conserved residues within the subgroup are in black, while semi-conserved residues are in white. Catalytic residues are underlined and include the oxyanion-hole Arg in addition to the classical triad Ser, His and Glu/Asp. The catalytic acid differs in the two subgroups. The corresponding sequence in the bacterial *Ot*CE15A is shown, which contains functional acid residues at both canonical and noncanonical positions. Coloured boxes correspond to the colours of the secondary-structure elements in (*b*). The correspondence of residues is based on structural alignment. (*b*) Selected structural elements of GEs illustrated with the structure of *Ot*CE15A (PDB code 6t0i). β5–β8 denote the main β-strands numbered according to the core ABH numbering. αL is an α-helix-containing loop involved in substrate binding. The semitransparent cyan surface shows the position of the product XU^2^X [2^2^-(4-*O*-methyl-α-d-glucuronyl)-xylotriose, also referred to as XUX]. (*c*) Overview of GE and other CE model substrates tested in this work. In BnzGlcA and MeGlcA, *R*_2_ is H and *R*_1_ is a benzyl or methyl group, respectively. In 4-*O-*Me-MeGlcA, both *R*_1_ and *R*_2_ are methyl groups.

Glucuronyl esterases (GEs) are carbohydrate-active enzymes that are able to cleave ester linkages between the alcohols of the aromatic polymer lignin and 4-*O*-methylglucuronic acid (4-*O-*MeGlcA) moieties on glucuronoxylan in the plant cell wall, a linkage which contributes to the recalcitrance of plant biomass (Špániková & Biely, 2006 ▸; Weng *et al.*, 2008 ▸). GEs are classified into carbohydrate esterase (CE) family 15 (CE15) in the carbohydrate-active enzyme database (https://www.cazy.org/; Drula *et al.*, 2022 ▸). Many biomass-degrading organisms (both bacteria and fungi) encode at least one gene from CE15, suggesting that these enzymes are necessary to efficiently degrade recalcitrant structures such as lignin carbohydrate complexes (LCCs). It has been proposed that the 4-methoxy group on the glucuronic acid is crucial for GE activity (Špániková & Biely, 2006 ▸; d’Errico *et al.*, 2015 ▸; Špániková *et al.*, 2007 ▸), although a lack of this decoration does not seem to hinder the hydrolysis of model substrates by a range of bacterial and fungal GEs (Arnling Bååth *et al.*, 2018 ▸; Hüttner *et al.*, 2017 ▸). Furthermore, the substrate profiles of GEs on model substrates (examples in Fig. 1 ▸) show variations, with some bacterial GEs acting on both glucuronoyl and galacturonoyl esters and having different preferences depending on the nature of the acyl group (Arnling Bååth *et al.*, 2018 ▸). Activity of GEs on substrates that are more similar to natural LCCs has been demonstrated on extracted LCCs (Arnling Bååth *et al.*, 2016 ▸) and, more recently, an LC-MS assay using a lignin-rich pellet (LRP) from birch as a substrate clearly showed GE activity of four fungal CE15 enzymes (Mosbech *et al.*, 2018 ▸).

Cip2 from *Trichoderma reesei*, a GE that has been shown to be important for the efficient hydrolysis of pre-treated corn stover (Lehmann *et al.*, 2016 ▸), was the first GE to be structurally characterized (Pokkuluri *et al.*, 2011 ▸). Interactions with a small model substrate have been structurally elucidated for *St*GE2 from *Thermothelomyces thermophiles* (Charavgi *et al.*, 2013 ▸). Several more experimental structures have since been obtained, totaling eight structures (three from fungal species and five from bacterial species) from diverse organisms. GEs belong to the α/β hydrolase (ABH) superfamily, with a catalytic triad common to serine hydrolases (Nardini & Dijkstra, 1999 ▸) consisting of a Ser nucleophile, a basic His residue and an acidic Glu/Asp residue (Fig. 1 ▸). Although the Ser and His residues are fully conserved amongst CE15 GEs, the location of the acidic residue differs within the family (Arnling Bååth *et al.*, 2019 ▸; De Santi *et al.*, 2017 ▸). Many bacterial GEs have an acidic residue in the canonical ABH position after β-strand 7 (Nardini & Dijkstra, 1999 ▸), while most fungal enzymes contain a Glu residue at a noncanonical position after β-strand 6. Some GEs, such as *Ot*CE15A from the soil bacterium *Opitutus terrae* (Fig. 1 ▸), have acidic residues at both positions; both residues have been shown to be involved in catalysis via biochemical/mutational studies and have more recently been further investigated using QM/MM calculations (Mazurkewich *et al.*, 2019 ▸; Zong *et al.*, 2022 ▸). Thorough structural characterization of the substrate-binding site of *Ot*CE15A revealed a number of different residues that are responsible for substrate binding and substrate stabilization (Mazurkewich *et al.*, 2019 ▸), and showed direct interaction with the main chain as well as the glucuronic acid moiety of a glucurono-xylooligosaccharide for the first time. This work was shortly followed by a similar characterization of substrate interaction of the fungal *Cerrena unicolor Cu*GE (Ernst *et al.*, 2020 ▸), in which the additional subdivision of CE15 into CE15-A and CE15-B was suggested based on positioning of the catalytic acid in the canonical or noncanonical position, respectively, identifying sequence signatures for the two structures (Fig. 1 ▸). Note that in Ernst *et al.* (2020 ▸), due to additional secondary-structure elements at the N-terminus of many GEs, the strand bearing the canonical position is denoted β8 and that bearing the noncanonical position is denoted β7, while here we denote the strands according to the common ABH core.

In a study characterizing several putative fungal GEs, some enzymes were inactive on model substrates despite being well expressed and apparently stable (Hüttner *et al.*, 2017 ▸). Similar to most other studied fungal GEs, these apparently inactive enzymes contain the catalytic serine and histidine residues and have the catalytic acid at the noncanonical position, as in the CE15-B subgroup. However, one of the putative GEs from *Lentithecium fluviatile*, *Lf*CE15C (formerly denoted LfGE3 in Hüttner *et al.*, 2017 ▸), lacks many of the additional sequence characteristics of a fungal CE15-B as described in Ernst *et al.* (2020 ▸). As highlighted in Fig. 1 ▸(*a*), a highly conserved glutamate residue in a substrate-interacting helix-containing loop (here denoted αL) is a glycine in *Lf*CE15C, while a conserved substrate-interacting tryptophan is instead a tyrosine. Thus, *Lf*CE15C, which is encoded as a single CE15 domain, was selected for further biochemical and structural investigation to explore the consequences of the residue differences and their potential impact on enzyme function.

## 2. Materials and methods

### 2.1. Sequence analysis

The genome of *L. fluviatile* was analysed by downloading all of its protein-coding sequences from NCBI, followed by the prediction of carbohydrate-active enzymes (CAZymes) using the *dbCAN*2 metaserver (https://bcb.unl.edu/dbCAN2/; Zhang *et al.*, 2018 ▸). For analysis of residue conservation not fitting into the CE15-A and CE15-B classifications, the sequence VN**G**DSWFSTDFSKYVDTVPTLPWDNHMLHALYAYPPRGLLIIENTAID**Y**LGPTSN containing the deviating G and Y (in bold) was used for a *BlastP* search, retrieving 99 sequences (including the query): 26 with G at the third position and 62 with E at the third position. Sequence logos were produced based on alignment of all of the retrieved sequences and the two subgroups using the *WebLogo* server at https://weblogo.berkeley.edu/logo.cgi.

### 2.2. Protein expression and purification

The CE15-C gene of *L. fluviatile* CBS 122367 (*Lf*CE15C, JGI protein ID Lenfl1|349146, GenBank KAF2678018.1) was codon-optimized for expression in *Pichia pastoris* and synthesized (NZYTech, Portugal) as described previously (Hüttner *et al.*, 2017 ▸). The construct contained the genomic sequence devoid of its predicted signal peptide-coding region. Briefly, the gene was cloned into pPICZα in-frame by EcoRI and XbaI restriction sites to include the N-terminal α-factor signal peptide and the C-terminal c-Myc epitope and His_6_ tag. The construct was genome-integrated into *P. pastoris* strain SMD1168H for protein production. The protein was purified on an ÄKTA system (Cytiva) in two steps. In the first step the protein was purified by immobilized metal-affinity chromatography (IMAC) on a 5 ml HisTrap Excel column using 50 m*M* Tris pH 8 with 250 m*M* NaCl as the binding buffer and a linear gradient of the same buffer containing 250 m*M* imidazole. Elution fractions were concentrated by ultrafiltration (Amicon Ultra-15, Merck–Millipore). In the second step (gel filtration), concentrated IMAC fractions were resolved on a HiLoad Superdex 200 16/60 column using the IMAC binding buffer as solvent. Protein samples were again concentrated by ultrafiltration and stored at 4°C.

The N241A, G254E, Y300W and G254E/Y300W substitution variants of *Lf*CE15C were created by site-directed mutagenesis using the QuikChange method (Liu & Naismith, 2008 ▸) and produced in *P. pastoris* SMD1168H as for the wild-type protein. All constructs and gene mutations were verified by DNA sequencing. Primer sequences utilized for mutagenesis are provided in Supplementary Table S1. Macromolecule-production information is summarized in Table 1 ▸.

### 2.3. Enzyme assays

Activity towards model substrates (Fig. 1 ▸ *c*) was tested at room temperature at three different pH values, 5.5, 6.5 and 7.5, in 0.1 *M* three-component constant ionic strength buffer consisting of 0.1 *M* Tris, 0.05 *M* acetic acid and 0.05 *M* MES (Ellis & Morrison, 1982 ▸; Mazurkewich *et al.*, 2016 ▸). Continuous spectrophotometric assays for GE activity were performed as described previously (Arnling Bååth *et al.*, 2018 ▸) by coupling d-glucuronate or d-galacturonate production to NADH oxidation by uronate dehydrogenase (Megazyme, Ireland). The uronic acid esters tested (Fig. 1 ▸ *c*) included benzyl glucuronate (BnzGlcA; 25 m*M*), methyl glucuronate (MeGlcA; 10 m*M*), 4-*O*Me-MeGlcA (5 m*M*) and methyl galacturonate (MeGalA; 10 m*M*). 4-*O*Me-MeGlcA was a kind gift from Professor P. Biely, while all others were purchased from Biosynth (previously CarboSynth). Feruloyl esterase and acetyl esterase activities were screened spectrophoto­metrically with methyl ferulate (MFA, 0.25 m*M*) and *p*NP-acetate (*p*NP-Ac, 10.0 m*M*), respectively, as described previously (Bonzom *et al.*, 2019 ▸; Kmezik *et al.*, 2020 ▸). Activity on *p*NP-[2^2^-(4-*O*-methyl-α-d-methylglucopyranosyluronate]-β-d-xylobioside was assayed using the K-GEUX3 coupled enzyme assay as described by the manufacturer (Megazyme). Briefly, activity was spectrophotometrically detected by measuring the absorbance at 400 nm after 10 min of incubation with the enzyme mixture in 0.1 *M* sodium phosphate buffer pH 6.5, 0.02%(*w*/*v*) sodium azide at 40°C. *Lf*CE15C concentrations in the range 8.7 n*M*–8.7 µ*M* were used.

Biomass saccharification-boosting assays to investigate potential increases in the monosaccharides released from the enzyme cocktail Ultraflo (Novozymes, Denmark) were performed similarly as described previously (Arnling Bååth *et al.*, 2018 ▸). Briefly, 2 ml hydrolysis reactions containing 1%(*w*/*v*) ball-milled corn cob and 0.1 mg Ultraflo (Novozymes, Denmark) per gram of dry weight, without or supplemented with 1 µ*M* *Lf*CE15C, were performed in triplicate in 25 m*M* sodium phosphate pH 6.0 at 25°C with vertical rotation. Reactions were stopped after 10, 30 or 60 min or overnight by heating at 95°C for 2 min. Debris was removed by centrifugation and the released monosaccharides were monitored by high-performance anion-exchange chromatography with pulsed amperometric detection on an ICS3000 system using a 4 × 250 mm Dionex Carbopac PA1 column with a 4 × 50 mm guard column maintained at 30°C (Dionex, Sunnyvale, California, USA). 25 µl samples were injected. The eluents were *A*, water; *B*, 300 m*M* sodium hydroxide; *C*, 100 m*M* sodium hydroxide, 85 m*M* sodium acetate. The samples were eluted isocratically with 100% eluent *A* for 40 min (1 ml min^−1^) and were detected by post-column addition of solvent *B* at 0.5 ml min^−1^. Peak analysis was performed using the *Chromeleon* software and the peaks were quantified against monosaccharide standards.

An additional boosting assay with destarched wheat bran (DWB; from ARD Pomacle France as in Bouraoui *et al.*, 2016 ▸) as a substrate was also performed using the enzyme cocktail Viscozyme (Novozymes, Denmark) together with *Lf*CE15C. The DWB was finely milled and 1 mg of the substrate was solubilized in 50 µl 0.1 *M* sodium acetate pH 5.5 in 1.5 ml test tubes. 10 µl buffer stock solution (0.5 *M* sodium acetate pH 5.5) was used to keep the salt concentration and the pH equivalent in all test tubes. Either 10 µl 0.6 µ*M* *Lf*CE15C, 10 µl 0.5 U Viscozyme or both were added to the test tubes. Milli-Q water was added to a total volume of 100 µl and the reactions were incubated on a thermoshaker at 50°C and 1000 rev min^−1^. After 3 min, 1, 2, 3 or 4 h the tubes were centrifuged at 3000 rev min^−1^ for 10 min to remove the insoluble substrate and 50 µl of the supernatant was added to 100 µl 3,5-dinitrosalicylic acid (DNSA) and boiled for 10 min at 95°C. The tubes were then centrifuged for 5 min at 3000 rev min^−1^ and 100 µl was transferred to a 96-well plate to measure the absorbance of the reduced form of DNSA at 540 nm to quantify the amount of reducing sugar ends (Miller, 1959 ▸).

### 2.4. Differential scanning fluorimetry (DSF)

The thermostability of *Lf*CE15C was assayed in different buffers by nanoDSF using a Tycho NT.6 (NanoTemper) in capillaries (NanoTemper). The device was set to measure the intrinsic fluorescence ratio (330/390 nm) of the protein when increasing the temperature (from 35 to 95°C over 3 min). Protein samples with a concentration of 1 mg ml^−1^ were used to measure the inflection point of the melting curve unless otherwise stated. Data were analysed with the instrument’s software. The buffers tested included 0.1 *M* sodium acetate pH 4.5, 0.05 *M* sodium acetate pH 5.5, 0.1 *M* sodium citrate pH 5.0, 0.1 *M* MES pH 6.0, 0.1 *M* sodium phosphate pH 6.5, 0.1 *M* HEPES pH 7.5 and 0.02 *M* Tris pH 8.0.

Furthermore, nanoDSF was used to measure thermal shifts after the addition of potential ligands at 10 and 20 m*M* concentration. The ligands included neutralized GlcA (pH 7), cellobiose (both from Sigma–Aldrich), xylooligosaccharides {xylobiose, xylotriose, xylotetraose and XU^2^XXr [2^3^-(4-*O*-methylglucuronyl)-α-d-xylotetraitol, also referred to as XUXXr], from Megazyme}, BnzGlcA and corn cob xylan (both from Biosynth, previously Carbosynth).

### 2.5. Crystallization and structure determination

Screening for crystallization was carried out by the sitting-drop vapour-diffusion method set up by an Oryx8 robot (Douglas Instruments) using 0.3 µl drops with a 3:1 or 1:1 protein solution:reservoir solution ratio (for additional details, see Table 2 ▸). Several crystal hits were obtained in the JCSG+ screen (Molecular Dimensions) at 4°C. The crystals were mounted in cryoloops at 4°C and frozen by plunging them into liquid nitrogen with no addition of cryoprotectant. Two conditions, denoted conditions *A* and *B* in Table 2 ▸, resulted in diffraction data (BioMAX, MAX IV, Lund, Sweden) suitable for structure determination.

Data for the first crystal were processed with *XDS*/*XSCALE* (Kabsch, 2010 ▸) manually, while data for the second crystal were processed by the automatic processing pipeline at BioMAX also utilizing *XDS*/*XSCALE*. Space group, unit-cell parameters and statistics for the collected data are shown in Table 3 ▸.

A preliminary structure was determined by molecular replacement with *MOLREP* (Vagin & Teplyakov, 2010 ▸) from the *CCP*4 suite (Winn *et al.*, 2011 ▸) using the structure of Cip2 (Pokkuluri *et al.*, 2011 ▸) from *Trichoderma reesei* (PDB entry 3pic) as a search model (51% sequence identity over 92% of the sequence) against the data from crystal *B*, which has a smaller asymmetric unit. A clear solution with two molecules in the asymmetric unit was obtained. The protein was manually modelled in *Coot* (Emsley *et al.*, 2010 ▸) by changing the amino acids in the template to those of *Lf*CE15C, followed by several rounds of restrained refinement in *REFMAC* (Vagin *et al.*, 2004 ▸) alternating with manual rebuilding. In the later stages N-glycosylation was modelled according to the electron density, which resulted in a preliminary structure in an orthorhombic space group with an *R*_free_ of 27.8%. This partially refined crystal *B* model was used as a model for the *P*1 data from crystal *A* (four molecules in the asymmetric unit) and further refined, including the addition of solvent molecules and extensive glycosylation at Asn241, for which the electron density was not very well defined. Two *cis*-Pro residues are found in the structure (115 and 286). NCS restraints were used during refinement. Final refinement and validation statistics are shown in Table 4 ▸. The structure of crystal *A* was deposited as PDB entry 8b48. 4–5 N-terminal residues from the mature protein (starting at residue 17 to match the native sequence including the native signal peptide) are missing from the model. The structure has very good geometry as judged from agreement with ideal bond/angle values, Ramachandran statistics and other geometric parameters, while the *R* factors are below average, probably owing to the extensive glycosylation which cannot be accurately modelled. Structures were visualized with *PyMOL* (version 1.7.7.0; Schrödinger).

## 3. Results and discussion

### 3.1. Sequence analysis of the *L. fluviatile* genome

Given the previously reported absence of activity towards BnzGlcA (Hüttner *et al.*, 2017 ▸) for all proteins corresponding to CE15 genes found in the *L. fluviatile* genome, it is pertinent to address whether *L. fluviatile* is expected to be a lignocellulose degrader possessing active GEs or whether the CE15 sequences represent proteins that have evolved for a different function. Descriptions of the habitat of the species are scarce, although isolation from dead wood material has been reported (https://www.gbif.org/occurrence/3128715977). Furthermore, no information is available in the literature on gene expression by *L. fluviatile* upon growth on lignocellulose. To further investigate the lignocellulose-degrading capacity of this fungus, its genome was analysed using the *dbCAN*2 server to predict its CAZyme repertoire. The prediction revealed a plethora of putative CAZymes, 641 in total, with 553 assigned to degradative classes (*i.e.* not glycosyl transferases). Based on this information, it appears that *L. fluviatile* could have the capacity to deconstruct most major constituents of plant biomass, with multiple putative enzymes from families commonly associated with lignocellulose degradation (Table 5 ▸). With this presumed ability to target both polysaccharides and lignin, including a large number of putative xylan-active enzymes, it can reasonably be expected that *L. fluviatile* also would possess active GEs among its proteins from CE15.

### 3.2. *Lf*CE15C is devoid of detectable GE activity

Based on the genome analysis, and the fact that GE activity is the only enzymatic activity consistently reported in CE15 to date, the purified *Lf*CE15C was expected to be active towards a variety of GE model substrates (Fig. 1 ▸) used previously (Arnling Bååth *et al.*, 2018 ▸). However, at the concentrations tested no activity was detectable for 15 min at room temperature for BnzGlcA (previously tested in Hüttner *et al.*, 2017 ▸), MeGlcA, MeGalA or 4-*O*-Me-MeGlcA, which has an additional methyl group that has been reported to be important for the activity of some fungal GEs (Ďuranová *et al.*, 2009 ▸). *Lf*CE15C was also devoid of ferulic acid esterase activity, assayed using MFA, and only trace activity was found with the generic *p*NP-Ac substrate, although this could be attributed to trace imidazole buffer remaining after purification giving rise to non-enzymatic hydrolysis. Furthermore, no activity could be detected in a coupled assay utilizing a slightly larger substrate GEUX3 consisting of a *p*NP-xylobioside backbone decorated with 4-*O-*Me-MeGlcA (Fig. 1 ▸).

Additional attempts were made to measure the boosting of the activity of known cellulolytic cocktails (Ultraflo and Viscozyme) on biomass. Boosting by *Lf*CE15C could not be detected under the given conditions either on corn cob biomass, where GE boosting of the Ultraflo cocktail with bacterial GEs has previously been demonstrated (Arnling Bååth *et al.*, 2018 ▸), or on DWB with Viscozyme.

### 3.3. *Lf*CE15C is a well folded protein with a typical α/β-hydrolase active site

**Figure 2:**
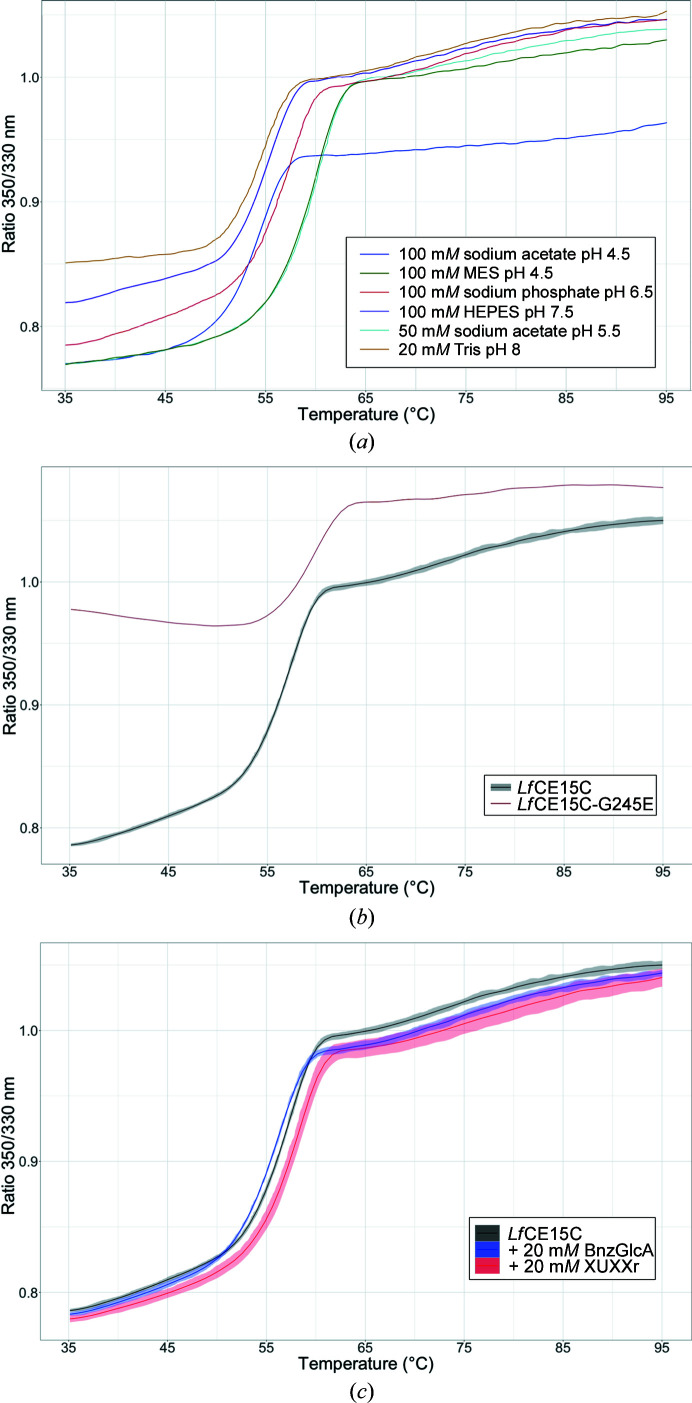
Representative nanoDSF unfolding curves for *Lf*CE15C. (*a*) Individual unfolding curves of *Lf*CE15C-wt in different buffers. (*b*) Average unfolding curve for *Lf*CE15C-wt and *Lf*CE15C-G254E in 0.1 *M* sodium phosphate buffer. (*c*) Average unfolding curves of *Lf*CE15C with either 20 m*M* XUXXr or BnzGlcA added to 0.1 *M* sodium phosphate buffer pH 6.5. The shaded region of the curves represents the standard deviation of three measurements.

As activity could not be detected on any of the tested substrates, it could be questioned whether *Lf*CE15C was in a properly folded state. NanoDSF measurements (Fig. 2 ▸ and Supplementary Table S2) showed a clear inflection point at ∼55°C even after storage for several months at 4°C in 20 m*M* Tris buffer pH 8.0, indicating a correctly folded protein. Further investigation shows that the thermal stability of the protein is highly buffer dependent and differing inflection points could be detected for the protein (Fig. 2 ▸ *a*). The more stabilizing buffers were 0.05 *M* sodium acetate pH 5.5 and 0.1 *M* MES pH 6.0, with *T*_i_ values of 60.2 and 59.9°C, respectively.

To investigate whether local structural features could shed light on the lack of activity, we determined the structure of *Lf*CE15C by X-ray crystallography. The structure was determined to a maximum resolution of 2.65 Å (crystal form *A*) with good overall geometry. The final model contains four protein chains, each with an N-glycosylation site at Asn241 modelled with variable number of carbohydrate units.

**Figure 3:**
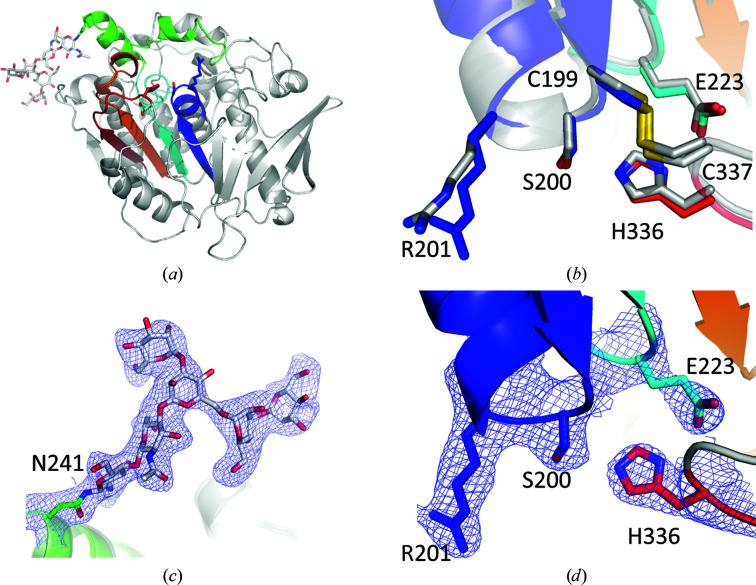
Structure of *Lf*CE15C. (*a*) Overall structure (chain *C*) using the same colour scheme as in Fig. 1 ▸. The active-site residues Ser200, Arg201, Glu223 and His336 and glycosylation at Asn241 are shown as sticks. (*b*) Active site of *Lf*CE15C overlaid with Cip2 (PDB entry 3pic, grey) with the residues from *Lf*CE15C labelled. One of the disulfide bridges is also shown. (*c*, *d*) Electron density at (*c*) the glycosylation site and (*d*) the active site of *Lf*CE15C chain *C* showing the 2*F*_obs_ − *F*_calc_ electron density contoured at 1.0σ.

The overall structure of *Lf*CE15C is defined by a three-layer αβα sandwich typical of the α/β-hydrolase fold and CE15 enzymes (Fig. 3 ▸ *a*). As expected from the sequence identity of over 50%, the structure is quite similar overall to Cip2 from *Hypocrea jecorina* (*T. reesei*), which was used as a molecular-replacement model (PDB entry 3pic; assigned as a CE15-B protein), with a C^α^ r.m.s.d. of 0.96 Å for 356 aligned residues. As seen in other fungal members of CE15, *Lf*CE15C is stabilized by several disulfide bonds (Cys21–Cys56, Cys199–Cys337 and Cys231–Cys309).

The catalytic triad consists, as expected, of the nucleophile Ser200 on the so-called ‘nucleophilic elbow’ at the end of β-strand 5, the acid Glu223 at the end of β-strand 6 typical of the CE15-B subgroup and His336 on a loop following β-strand 8 (Figs. 1 ▸ *a* and 3 ▸). All catalytic residues have conformations similar to those in previously determined structures of CE15 proteins, exemplified in Fig. 3 ▸(*b*) by the Cip2 structure. The active-site structure is stabilized by one of the aforementioned disulfide bonds (Cys199–Cys337), also conserved in Cip2, that joins the strand bearing the serine nucleophile to the loop bearing the catalytic histidine. In many ABHs the oxyanion hole facilitating the charge stabilization of the transition state consists exclusively of main-chain N atoms. However, in CE15 GEs an Arg side chain immediately following the catalytic serine (Arg201 in *Lf*CE15A) is found to fulfil this role, as recently investigated in detail (Zong *et al.*, 2022 ▸), and thus the catalytic machinery of *Lf*CE15A is fully consistent with a functional GE enzyme. Furthermore, the glycosylation, which may be non-native due to expression in *P. pastoris*, points away from the active site and is thus is unlikely to interfere with the catalytic activity (Fig. 3 ▸ *a*).

**Figure 4:**
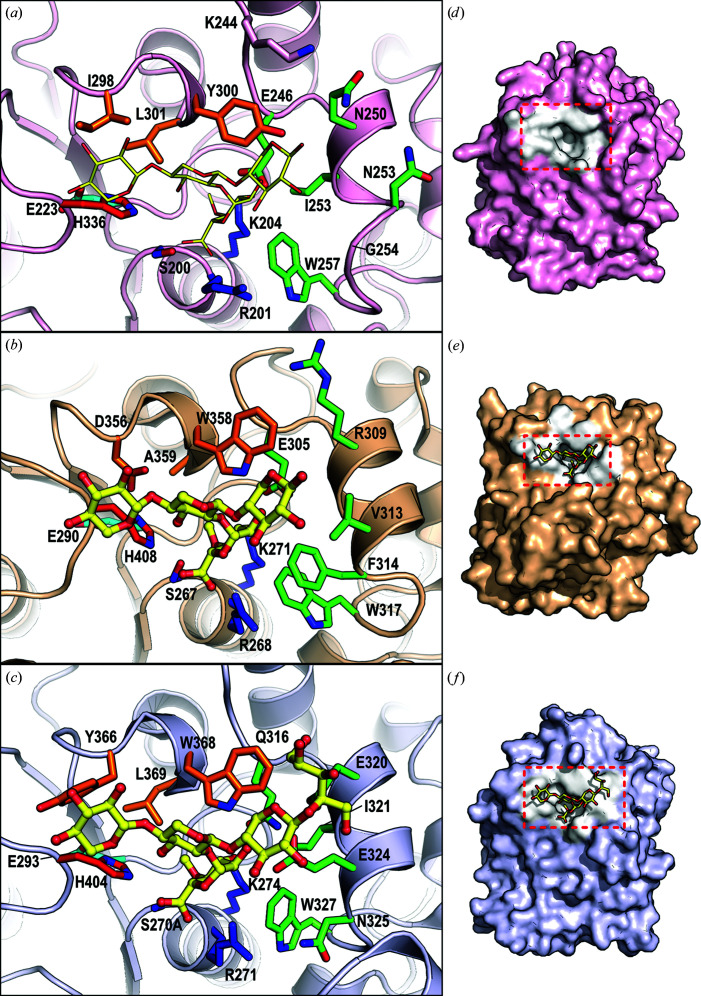
Comparison of the active sites of selected CE15 enzymes. The active site of (*a*) *Lf*CE15C (with superposed XUX from PDB entry 6t0i) is compared with the active sites of (*b*) *Ot*CE15A (PDB entry 6t0i) and (*c*) *Cu*GE (PDB entry 6rv9) crystallized with XUX and XUXXr, respectively. Catalytic and substrate-interacting residues are shown as sticks and are colour-coded as in Fig. 1 ▸. (*d*), (*e*) and (*f*) are the corresponding surface views, with binding residues in white. The binding pockets are emphasized by a dashed square. In other GEs there are larger residues in the corresponding position to Gly254 in *Lf*CE15C, which in the latter creates a larger cavity that is capable of accommodating additional xylan decorations (Fig. 5 ▸).

As exemplified by the structures of *Ot*CE15A and *Cu*GE in complex with plant cell-wall oligosaccharides (Figs. 4 ▸ *b* and 4 ▸ *c*; Mazurkewich *et al.*, 2019 ▸; Ernst *et al.*, 2020 ▸), a conserved lysine in the helix immediately following β-strand 5 (Fig. 1 ▸ *a*) interacts with O3 on the 4-*O-*Me-GlcA moiety of the substrate, and a conserved tryptophan residue from αL (an α-helix-rich loop; green in Figs. 1 ▸ and 3 ▸) interacts with the carbohydrate ring (Figs. 4 ▸ *b* and 4 ▸ *c*). Both residues are conserved in *Lf*CE15C (Lys204 and Trp257).

As expected from the previous sequence analysis, some of the residues responsible for forming the expected substrate-binding pocket do not conform to previously determined structures of active GEs or the CE15-B sequence signature. In the αL region the glutamine observed to interact with O2 and O3 in *Cu*GE (Gln316 in *Cu*GE) is a glutamate in *Ot*CE15A (Glu305) and in fungal CE15-A members, but also in the CE15-B member *Lf*CE15C (Glu246). The glutamate residue can presumably be functionally equivalent to glutamine, so this difference is unlikely to be of functional importance. In contrast, the characteristic glutamate of fungal CE15-B (Fig. 1 ▸ *a*) further along in the αL region (Glu324 in *Cu*GE), which interacts with O2 of the GlcA moiety as well as the xylan backbone, is substituted by a glycine in *Lf*CE15C (Gly254). This is a major deviation from the proposed sequence signature of CE15-B, conforming more to fungal CE15-A, where the residue is often a glycine. In bacterial GEs such as *Ot*CE15A this glutamate is not conserved (Val313 in *Ot*CE15A). In both cases, however, the size of the binding pocket is smaller than in *Lf*CE15C owing to the presence of residue side chains at this location (Figs. 4 ▸ *d*–4 ▸ *f*).

Furthermore, an otherwise extremely conserved tryptophan in the whole CE15 family (Trp358 in *Ot*CE15A and Trp368 in *Cu*GE and CE15-B, phenylalanine or tryptophan in fungal CE15-A), which interacts with GlcA O2 and is located at the end of β-strand 7 in the loop following the canonical acid residue position, is found to be a tyrosine in *Lf*CE15C. Although in principle this is a conservative substitution, the hydrogen bond between the NH group of tryptophan and O2 of the GlcA moiety will almost certainly be lost given the conformation of the corresponding tyrosine in the active site. Thus, while *Lf*CE15C has the typical catalytic machinery expected of an active GE, it has a distinct and wider binding site, which could perhaps accommodate additional side chains from hemicellulose and/or be the cause of the lack of activity with the model substrates described above.

### 3.4. Residue substitution does not result in activity on model substrates

As the major differences in the substrate-binding site of *Lf*CE15C compared with GEs with demonstrated activity on model substrates are a tyrosine-to-glycine and a tryptophan-to-tyrosine substitution, we produced G254E, Y300W and G254E+Y300W variants. Additionally, to probe whether glycosylation at Asn241 could indirectly affect the enzymatic activity, although no interference is suggested by the structure, we produced an N241A variant. Activity on model substrates was tested on all variants as for the wild-type (wt) enzyme shortly after protein production, but again no activity of any of the variants could be detected. The G254E variant was shown to have a similar long-term stability to the wt enzyme as shown by the *T*_i_ measured several months after purification (Fig. 2 ▸ *b* and Supplementary Table S2); thus, the lack of activity cannot be attributed to a lack of stability.

### 3.5. Thermal shift analysis is compatible with *Lf*CE15C binding LCC fragments

Although activity on more complex substrates cannot easily be tested for *Lf*CE15C due to the lack of suitable pure compounds to test, we hypothesized that thermal shift assays might detect the binding of cell-wall fragments, as previously shown for *Ck*GE15 (Krska *et al.*, 2021 ▸). Initially, this was tested in 20 m*M* Tris buffer pH 8.0 with ligands at 10 m*M*, which resulted only in small thermal shifts and/or a change in the fluorescence ratio in the presence of XUXXr and BnzGlcA. We therefore increased the ligand concentration to 20 m*M* to see whether an increased effect could be detected, but this caused a pH shift due to the uronic acid. We therefore continued the thermal shift assays in 0.1 *M* sodium phosphate pH 6.5, which maintained the pH (and also increased the stability of *Lf*CE15C). A decrease in *T*_i_ was observed with BnzGlcA and an increase in *T*_i_ was observed with XUXXr, accompanied by changes in the initial fluorescence ratio (Fig. 2 ▸ *c* and Supplementary Table S2), which give an indirect indication of binding. To test our hypothesis that *Lf*CE15C needs additional xylan decorations for binding and activity, a similar experiment with a commercial (now discontinued) low-molecular-weight corn cob xylan was attempted, as this mixture was supposed to have both 4-*O*Me-GlcA and arabinofuranose substitutions on the xylan backbone. No thermal shift was detected, but subsequent mass-spectrometric analysis also showed that no (4-*O*Me)-GlcA was present as a substituent (not shown).

### 3.6. *Lf*CE15C is likely to be a GE with specificity for more complex substrates

**Figure 5:**
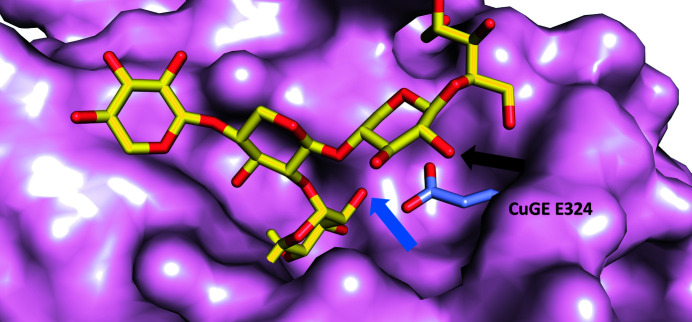
Close-up of the extra cavity in *Lf*CE15C where additional hemicellulose decorations could be accommodated. *Lf*CE15C is shown as a surface with the overlaid structure of *Cu*GE (PDB entry 6rv9). Only the bound XUXXr and Glu324 (a glycine in *Lf*CE15C) are shown for *Cu*GE. Possible attachment sites for additional decorations are indicated by the black arrow (O2 arabinose decoration on the xylan backbone) and blue arrow [a rare pentose decoration on GlcA as reported by Mortimer *et al.* (2015 ▸) and Peña *et al.* (2016 ▸)].

Despite the lack of activity on any GE substrate tested, the structure of *Lf*CE15C is typical of an active ABH, and the catalytic machinery in particular is structurally conserved compared with other GEs, strongly suggesting that *Lf*CE15C is an active enzyme. Furthermore, analysis of the genome of *L. fluviatile* supports the notion that it is a lignocellulose degrader, in which GE activity is to be expected. Evidence, albeit weak, for binding of biomass components by *Lf*CE15C was obtained in the form of small thermal shifts and changes in intrinsic fluorescence in the presence of XUXXr and BnzGlcA. The substrate-binding site has conserved elements, but also differs from other GEs, with additional cavities near the GlcA binding pocket in the active site (Figs. 4 ▸ and 5 ▸). Taken together, our work suggests activity on biomass containing hemicelluloses with a high degree of and/or unusual decorations. In particular, glucuronoxylans with a pentose decoration at the O2 of (4-*O*Me-)GlcA (Peña *et al.*, 2016 ▸; Mortimer *et al.*, 2015 ▸) would provide a good fit to the additional cavity (Fig. 5 ▸, blue arrow). Unfortunately, the lack of more natural model substrates, or even well defined complex uronic acid oligosaccharides, for binding studies precludes further investigation of the specificity of *Lf*CE15C at this stage. The lack of boosting ability on corn cob or wheat bran suggests that other biomass sources than grasses should be investigated in any future boosting studies. To date, pentose substitutions on GlcA have been reported for *Arabidopsis* primary cell wall (Mortimer *et al.*, 2015 ▸) and Asparagales and Alismatales species (Peña *et al.*, 2016 ▸).

**Figure 6:**
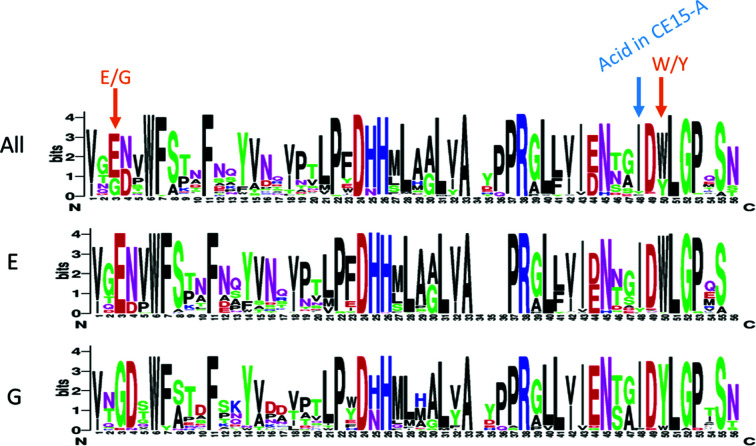
Sequence logos of sequences identified through a database search with part of the *Lf*CE15C sequence (see Section 2). The top shows the logo of all sequences, the middle the logo of the subset with glutamate at position 3 and the bottom the logo of the subset with glycine at position 3. Practically all sequences found have the isoleucine characteristic of CE15-B (see Fig. 1 ▸ *a*) at the position occupied by the acid in CE15-A (blue arrow), and thus can be assigned to CE15-B despite the unusual sequence features. The position of the tryptophan or tyrosine residue found to correlate with the presence of either a glutamate or glycine residue, respectively, is shown.

Another pertinent question is whether *Lf*CE15C is an isolated unusual enzyme or represents a subgroup with similar structural characteristics. Using a 56-residue sequence from *Lf*CE15C including both Gly254 and Tyr300 as a motif for a sequence-database search identified 99 sequences with a mixture of glutamate and glycine at position 3 corresponding to Gly254 (Fig. 6 ▸, top) and a mixture of tryptophan and tyrosine at the corresponding position to Tyr300. The sequence logos of subsets of sequence hits with glutamate or glycine at position 3 clearly show that glutamate correlates with tryptophan, while glycine highly correlates with tyrosine (Fig. 6 ▸, middle and bottom). This latter subgroup of >20 sequences, like *Lf*CE15C, has the catalytic acid glutamate at the end of β6 as typical of fungal CE15-B, instead of at the end of β7 as typical of CE15-A, but has the glycine typical of fungal CE15-A at position 3 instead of the conserved glutamate at the same position typical of CE15-B. The source organisms include fungal species from various environments (Supplementary Fig. S1). Thus, the unusual subset of CE15 enzymes represented by *Lf*CE15C has characteristics of both CE15-A and CE15-B, suggesting that this division is not as clear-cut as previously proposed (Ernst *et al.*, 2020 ▸). The glycine/tyrosine pair is most probably significant for substrate specificity rather than correlating with a specific catalytic machinery.

The substrate-binding site of *Lf*CE15C appears to be able to accommodate additional side chains compared with current protein–ligand structures of GEs. Although we have not yet been able to prove this, we suggest that *Lf*CE15C and other CE15 members in this subgroup may need substrates that contain larger hemicellulose portions to appropriately position the cleavable bond for catalysis/have sufficient affinity for substrate binding and may be needed for the degradation of rare xylan–lignin linkages found in specific plant cell walls.

## Supplementary Material

PDB reference: *Lf*CE15C, 8b48

Supplementary Tables and Figure. DOI: 10.1107/S205979832300325X/jc5055sup1.pdf

## Figures and Tables

**Table 1:** Macromolecule-production information

Source organism	*Lentithecium fluviatile* CBS 122367
DNA source	Synthesized
Cloning vector	pPICZα
Expression vector	pPICZα
Expression host	*Pichia pastoris* strain SMD1168H
Complete amino-acid sequence of the construct produced†	**MRFPSIFTAVLFAASSALAAPVNTTTEDETAQIPAEAVIGYSDLEGDFDVAVLPFSNSTNNGLLFINTTIASIAAKEEGVSLEKREAEA**EFQAPSCPNLPASINYAANPKLPDPFLALSGTRLSKKDQWPCRKEEIRQLFQRYSYGTFPPRPESVTAAMSGNALKITVSEGSKSMSFSVNIKLPSSGAAPYPAIIAYGSASLPIPNTVATITYQNFEMAADNGRGKGKFYEFYGSNHNAGGMIAAAWGVDRIIDALEMTPAAKIDPKRVGVTGCSRNGKGSMIAGAFVDRIALALPQEGGQSAAGCWRIADEIQKNGTKVETAHQIVNGDSWFSTDFSKYVDTVPTLPWDNHMLHALYAYPPRGLLIIENTAIDYLGPTSNYHCATAGRKVHEALGVKDYFGFSQNSHSDHCGFPKAQQPELTAFIERFLLAKDTKTDVWKTDGKFTIDERRWIDWAVPSLSGL*EQKLISEEDLNSAVDHHHHHH*

† The N-terminal α-factor signal peptide is underlined. The portion shown in bold is removed in the processed protein. The C-terminal c-Myc epitope and His tag are shown in italics.

**Table 2:** Crystallization conditions

Method	Vapour diffusion, sitting drop
Plate type	MRC 2-drop 96-well plate (Douglas Instruments)
Temperature (K)	277.15
Protein concentration (stock) (mg ml^−1^)	13.7
Buffer composition of protein solution	20 m*M* Tris pH 8.0
Composition of reservoir solution	*A*, 0.2 *M* ammonium formate pH 6.6, 20%(*w*/*v*) PEG 3350; *B*, 0.1 *M* potassium thiocyanate, 30%(*w*/*v*) PEG MME 2000
Volume and ratio of drop	0.3 µl, 3:1 protein stock:reservoir solution
Volume of reservoir (µl)	100

**Table 3:** Data collection and processing Values in parentheses are for the outer shell.

Crystal	*A*	*B*
Diffraction source	BioMAX, MAX IV	BioMAX, MAX IV
Wavelength (Å)	0.980779	0.980779
Temperature (K)	100	100
Detector	EIGER 16M	EIGER 16M
Crystal-to-detector distance (mm)	335.7	335.7
Rotation range per image (°)	0.10	0.10
Total rotation range (°)	400	400
Exposure time per image (s)	0.01	0.01
Space group	*P*1	*P*2_1_2_1_2_1_
*a*, *b*, *c* (Å)	70.74, 79.57, 86.01	79.70, 89.40, 91.93
α, β, γ (°)	113.32, 98.53, 94.44	90.0, 90.0, 90.0
Mosaicity (°)	0.266	0.201
Resolution range (Å)	47.21–2.65 (2.72–2.65)	30.0–3.11 (3.29–3.11)
Total No. of reflections	182290 (13800)	178934 (27911)
No. of unique reflections	47780 (3530)	12326 (1922)
Completeness (%)	97.4 (97.2)	99.5 (99.5)
Multiplicity	3.8 (3.9)	14.5 (14.5)
〈*I*/σ(*I*)〉†	4.3 (1.0)	9.7 (3.0)
*R*_r.i.m._ (%)	24.0 (125.0)	24.9 (80.6)
CC_1/2_ (%)	98.2 (51.3)	99.5 (92.1)
Overall *B* factor from Wilson plot (Å^2^)	65.5	60.5

† Although 〈*I*/σ(*I*)〉 is low in the outer resolution shell, CC_1/2_ > 50% clearly indicates that the data are usable at the highest given resolution.

**Table 4:** Structure solution and refinement for crystal form *A* Values in parentheses are for the outer shell.

Resolution range (Å)	47.254–2.650 (2.719–2.650)
Completeness (%)	97.6
σ Cutoff	None
No. of reflections, working set	45487 (3350)
No. of reflections, test set	2294 (182)
Final *R*_work_	0.238 (0.455)
Final *R*_free_	0.298 (0.484)
ESU based on maximum likelihood (Å)	0.509
No. of non-H atoms	
Protein	11407
Glycosylation	244
Water	303
Formate	6
R.m.s. deviations	
Bond lengths (Å)	0.006
Angles (°)	1.448
Average *B* factors (Å^2^)	
Protein	62.8
Glycosylation	116.8
Water	37.2
Formate	67.1
Ramachandran plot†	
Favoured (%)	94.1
Outliers (%)	0.0
*MolProbity* score†	2.08

† Calculated using *MolProbity* (https://molprobity.biochem.duke.edu/; Williams *et al.*, 2018 ▸).

**Table 5:** Analysis of putative CAZymes in the genome of *L. fluviatile* Listed are the predicted members from glycoside hydrolase (GH), carbohydrate esterase (CE), auxiliary activities (AA), and polysaccharide lyase (PL) families, with family number indicated. The number in parenthesis shows the number of identified modules from each family.

Putative substrate	CAZy family and number of modules
Cellulose	GH3 (15), GH5 (19), GH6 (4), GH7 (5), AA9 (52), AA16 (3)
Xylan	GH10 (5), GH11 (4), GH30 (2), GH43 (15), GH51 (2), GH62 (2), GH67 (1), GH115 (2), CE1 (9), CE3 (4), CE4 (8), CE5 (11), CE15 (3), AA14 (1)
Mannan	GH26 (1), GH27 (6)
Pectin	GH28 (8), GH51 (1), GH78 (1), GH93 (2), PL1 (8), PL3 (6), PL4 (5), PL9 (1), PL26 (1)
Lignin	AA1 (8), AA2 (13)
