# Supplementary material for: Structural and functional investigation of a fungal member of carbohydrate esterase family 15 with potential specificity for rare xylans
Source: Acta Crystallogr D Struct Biol. 2023 May 25;79(Pt 6):545–55. doi: 10.1107/S205979832300325X (PMC10233622; doi:10.1107/S205979832300325X)
Supplement: Supplementary file 1 [file d-79-00545-sup1.pdf]

STRUCTURAL
BIOLOGY

Volume 79 (2023)

Supporting information for article:

Structural and functional investigation of a fungal member of carbohydrate esterase family 15 with potential specificity for rare xylans

Scott Mazurkewich, Karoline C. Scholzen, Rikke H. Brusch, Jens-Christian N. Poulsen, Yusuf Theibich, Silvia Hüttner, Lisbeth Olsson, Johan Larsbrink and Leila Lo Leggio

Table S1 Primers utilized in the generation of protein substitution variants. Introduced mutations are displayed in lower case.

*Lf*CE15C-N241A-r	AGTACCGgcCTTTTGGATTTCGTCAGCAATTC
*Lf*CE15C-N241A-f	CAAAAGgcCGGTACTAAAGTTGAGACAGCCC
*Lf*CE15C-G254E-r	GAATCTtCATTGACAATCTGGTGGGC
*Lf*CE15C-G254E-f	GTCAATGaAGATTCTTGGTTTTCCACAGACTTC
*Lf*CE15-Y300W-r	TGGTCCAAGccAGTCAATAGCGGTGTT
*Lf*CE15-Y300W-f	ATTGACTggCTTGGACCAACATCAAATTATCATTGTG

Table S2 nDSF measurements of *Lf*CE15C. Protein concentration was 1 mg/mL.

Protein	Ligand	Buffer	T_i in °C*	Initial fluorescence ratio
*Lf*CE15C	-	20 mM Tris pH 8.0	54.7±0.13 (5)	0.8498±0.0014
*Lf*CE15C	-	100 mM MES pH 6.0	59.9	0.7702
*Lf*CE15C	-	100 mM HEPES pH 7.5	55.2	0.8182
*Lf*CE15C	-	100 mM sodium citrate pH 5.0	48.7	0.7795
*Lf*CE15C	-	50 mM sodium acetate pH 5.5	60.2	0.7696
*Lf*CE15C	-	100 mM sodium acetate pH 4.5	54.4	0.7701
*Lf*CE15C-G254E	-	20 mM Tris pH 8.0	57.2	0,9591
*Lf*CE15C-G254E	-	100 mM sodium phosphate pH 6.5	59.6	0.9775
*Lf*CE15C	-	100 mM sodium phosphate pH 6.5	57.1±0.08 (3)	0.7861±0.0014
*Lf*CE15C	10 mM GlcA	20 mM Tris pH 8.0	54.8±0.18 (4)	0.8493±0.0011
*Lf*CE15C	20 mM GlcA	20 mM Tris pH 8.0	55.2±0.42 (4)	0.8463±0.0029
*Lf*CE15C	20 mM GlcA	100 mM sodium phosphate pH 6.5	57.2±0.04 (3)	0.7878±0.00022
*Lf*CE15C	10 mM BnzGlcA	20 mM Tris pH 8.0	55.9±0.67 (4)	0.8330±0.012
*Lf*CE15C	20 mM BnzGlcA	20 mM Tris pH 8.0	55.4±0.56 (4)	0.8529±0.0051
*Lf*CE15C	20 mM BnzGlcA	100 mM sodium phosphate pH 6.5	56.0±0.04 (3)	0.7835±0.0016
*Lf*CE15C	10 mM cellobiose	20 mM Tris pH 8.0	54.9±0.30 (4)	0.8480±0.0017
*Lf*CE15C	20 mM cellobiose	20 mM Tris pH 8.0	55.4±0.22 (4)	0.8452± 0.0021
*Lf*CE15C	20 mM cellobiose	100 mM sodium phosphate pH 6.5	57.4±0.07 (3)	0.7849±0.00032
*Lf*CE15C	10 mM xylobiose	20 mM Tris pH 8.0	54.9±0.16 (4)	0.8483±0.0012
*Lf*CE15C	20 mM xylobiose	20 mM Tris pH 8.0	55.4±0.28 (4)	0.8460±0.0024
*Lf*CE15C	20 mM xylobiose	100 mM sodium phosphate pH 6.5	57.2±0.08 (3)	0.7852±0.00031
*Lf*CE15C	10 mM xylotriose	20 mM Tris pH 8.0	54.8±0.25 (4)	0.8479±0.0017

*Lf*CE15C	20 mM xylotriose	20 mM Tris pH 8.0	55.3±0.25 (4)	0.8453±0.0026
*Lf*CE15C	20 mM xylotriose	100 mM sodium phosphate pH 6.5	57.3±0.12 (3)	0.7851±0.00032
*Lf*CE15C	10 mM xylotetraose	20 mM Tris pH 8.0	54.8±0.16 (4)	0.8499±0.0011
*Lf*CE15C	20 mM xylotetraose	20 mM Tris pH 8.0	55.2±0.25 (4)	0.8453 ±0.00090
*Lf*CE15C	20 mM xylotetraose	100 mM sodium phosphate pH 6.5	57.2±0.05 (3)	0.7856±0.0015
*Lf*CE15C	10 mM XUXXr	20 mM Tris pH 8.0	55.1±0.11 (4)	0.8481±0.00083
*Lf*CE15C	20 mM XUXXr	100 mM NaH_2PO_4 pH 6.5	58.0±0.25 (3)	0.7799±0.0022

* ± std. dev. for repeated measurements. The number of measurements is indicated in parentheses.

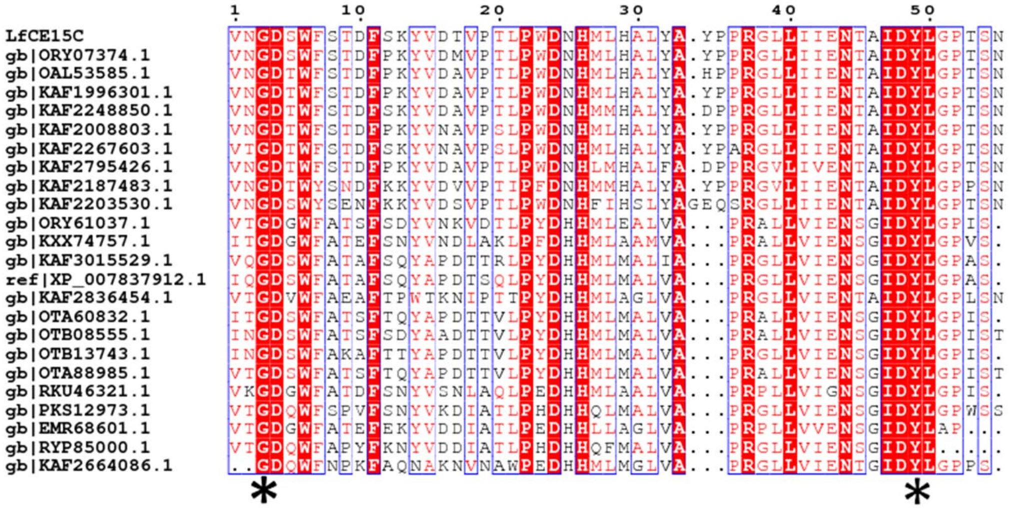

Figure S1 Alignment of found CE15 sequences containing G and Y at key positions in the region shown in Figure 6 (marked with an asterisk under the alignment. Genbank accession, species (habitat/isolation when known) are: ORY07374, *Clohesyomyces aquaticus* (submerged wood); OAL53585.1, *Pyrenochaeta* sp. DS3sAY3a (soil and plant debris); KAF1996301.1, *Amniculicola lignicola* CBS 123094 (submerged wood); KAF2248850.1, *Trematosphaeria pertusa* (submerged wood); KAF2008803.1, *Aaosphaeria arxii* CBS 175.79 (isolated from corn); KAF2267603.1, *Didymosphaeria enalia* (mangrove wood); KAF2795426.1, *Melanomma pulvis-pyrius* CBS 109.77 (debris of trees and shrubs); KAF2187483.1, *Zopfia rhizophila* CBS 207.26 (Zopfia root rot of asparagus); KAF2203530.1, *Delitschia confertaspora* ATCC 74209 (dung of rock hyrax); ORY61037.1, *Pseudomassariella vexata*; KXX74757.1, *Madurella mycetomatis* (human pathogen, mycetoma); KAF3015529.1, *Neopestalotiopsis* sp. 37M (fruit pathogen); XP_007837912.1, *Pestalotiopsis fici* W106-1 (plant endophyte); KAF2836454.1, *Patellaria atrata* CBS 101060 (canopy of floodplain forests); OTA60832.1, *Hypoxylon* sp. EC38 (*Neea floribunda shrub)*; OTB08555.1, Hypoxylon sp. CI-4A (*Persea indica endophyte);* OTB13743.1, *Daldinia* sp. EC12 (*Myroxylon balsamum endophyte)*; OTA88985.1, *Hypoxylon* sp. CO27-5 (mangrove); RKU46321.1, *Coniochaeta pulveracea* (decaying acacia); PKS12973.1, *Lomentospora prolificans* (opportunistic human pathogen); EMR68601.1, *Eutypa lata* UCREL1 (eutypa dieback of grapevines); RYP85000.1, *Monosporascus* sp. CRB-8-3 (arid plant roots); KAF2664086.1, *Microthyrium microscopicum* (tree leaves/stems).
